# Quantification of sugars and organic acids in tomato fruits

**DOI:** 10.1016/j.mex.2018.05.014

**Published:** 2018-05-25

**Authors:** Carlos Agius, Sabine von Tucher, Brigitte Poppenberger, Wilfried Rozhon

**Affiliations:** a Biotechnology of Horticultural Crops, TUM School of Life Sciences Weihenstephan, Technical University of Munich, Liesel-Beckmann-Straße 1, 85354 Freising, Germany; b Chair of Plant Nutrition, Department of Plant Sciences, TUM School of Life Sciences Weihenstephan, Technical University of Munich Emil-Ramann-Straße 2, 85350 Freising, Germany

**Keywords:** Quantification of sugars and organic acids by HPLC and GC–MS, Citric acid, Fructose, GC–MS, Glucose, HPLC, Malic acid, Solid phase extraction

## Abstract

Sugar and organic acid contents are major factors for tomato fruit flavour and are important breeding traits. Here we provide an improved protocol for accurate quantification of the main sugars, glucose and fructose, and the organic acids, citric acid and malic acid, present in tomato. The tomato extract is spiked with lactose and tricarballylic acid as internal standards and loaded onto a NH2 solid phase extraction (SPE) column. The sugars appear in the flow-through and are subsequently analysed by HPLC using a Nucleodur NH2 column and a refractive index detector. The organic acids bind to the SPE column and are eluted with 400 mM phosphoric acid. For analysis, the organic acids are separated by HPLC using a Nucleodur C18ec column and detected by UV absorption at 210 nm. The method shows excellent inter-day and intra-day reproducibility for glucose, fructose and citric acid with standard deviations of 1–5%. Quantification of citric acid by HPLC and GC–MS showed perfect agreement with a deviation of less than 3%.

• Simple method for quantification of glucose, fructose, citric acid and malic acid in tomato.
• Efficient removal of interfering compounds by solid phase extraction.
• High intra and inter-day reproducibility.

## Graphical abstract

**Figure:**
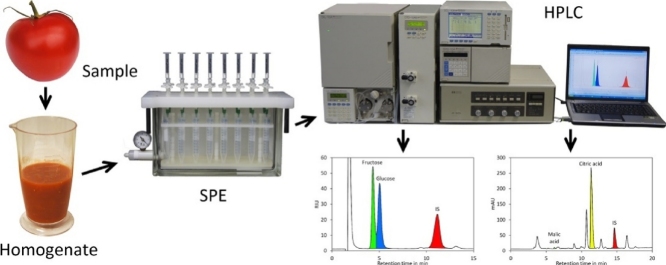

Specifications Table

**Table:** 

Subject area	Agricultural and Biological Sciences
More specific subject area	Metabolite analysis
Method name	Quantification of sugars and organic acids by HPLC and GC-MS
Name and reference of original method	*Quantification of sugars* Gancedo and Luh [12] Journal of Food Science 51, 571–573. Yelle et al. [13] Plant Physiology 90, 1473–1477. *Quantification of organic acids* Marconi et al. [27] Journal of Food Quality 30, 253-266. Manríquez et al. [21] Postharvest Biology and Technology 94, 58-65. Lee [20] Journal of Agricultural and Food Chemistry 41, 1991–1993. Shurubor et al. [22] Analytical Biochemistry 503, 8–10.
Resource availability	NA

## Method details

### Background

Sugar and acid contents are major factors for the flavour of tomato fruits and high but balanced levels of sugars and organic acids are desired. Both, the sugar and acid contents are important traits for breeding [[1], [2], [3], [4]]. Fruits of cultivated tomato (*Lycopersicon esculentum*) contain mainly glucose and fructose and only trace amounts of sucrose, while wild tomato species, for instance *Lycopersicon chmielewskii*, may contain sucrose as a main sugar [5]. The contents of sugars and organic acids of tomato fruits are highly dependent on the developmental stage and ripeness [6,7]. During ripening the total amount of sugars increases to approximately 4% with glucose being predominant in green, unripe fruits while red, fully ripe fruits contain typically slightly more fructose than glucose [5,7]. With increasing maturity after ripening the sugar content declines again [6]. The content of organic acids is also developmentally controlled and has been reported to increase during ripening [8]. At all stages citric acid is the dominant organic acid but unripe green tomatoes may contain significant amounts of malic acid while its content in ripe fruits is fairly low [9]. Similar to sugars, citric acid declines with progressing maturation after ripening while the content of malic acid remains relatively constant [6].

Tomatoes, as climacteric fruits, can ripen off-the-vine and it is a common commercial practise to harvest mature green or breaker stage (incipient red colour) fruits and to ripen them in transit or destination [3]. However, fruits ripened off-the-vine were shown to contain less sugars but similar levels of organic acids compared to fruits ripened attached to the mother plant [10,11], a difference that may negatively impact the flavour. Due to the importance of sugar and organic acid contents of tomato for breeding, quality assessment and physiological investigations a number of methods have been developed for quantification of these compounds.

Sugars were traditionally analysed by their capacity to reduce copper (II) or silver(I) ions. However, these methods were labour and time consuming and allowed only a rough differentiation of sugars in reducing and non-reducing sugars. Nowadays, mainly chromatographic [12,13], electrophoretic [14,15] and enzymatic methods are used [6,16,17] but also NMR [11], FTIR [18] and NIR [19] are applied. A convenient method for analysis of sugars includes separation on an amino (NH2) column with acetonitrile/water mixtures as eluent and detection using a refractive index (RI) detector [12,13]. Separation is based on interaction of the NH2 groups of the stationary phase with hydroxy groups of the sugars. Roughly, the more hydroxy groups a sugar has the stronger it interacts with the stationary phase and the later it elutes. Consequently, monosaccharides elute first, followed by disaccharides and trisaccharides. In addition to the number, also the position of hydroxy groups on the molecule is crucial for retention, thus allowing separation of different mono-, di- and trisaccharides. This method has the advantage that the sample can be directly loaded, no derivatisation steps are required and that amino columns are comparably cheap. However, organic acids and other compounds present in samples may bind strongly or even irreversibly to the column, which may influence retention and separation of sugars and reduce column lifetime.

Organic acids are frequently analysed in fruits, juices and other types of biological fluids by reversed phase (RP) HPLC [[19], [20], [21], [22]], ion exclusion chromatography [12,23], gas chromatography [24,25], enzymatic assays [16,17] and NMR spectroscopy [11,26]. For RP-HPLC aqueous acidic buffers containing no or small amounts of organic modifiers are used as eluents. Detection is possible by UV absorption at 210 nm. Since the carboxyl group is a weak chromophore detection is not very sensitive but sufficient for detection of the main acids in fruits. However, a more serious problem is the extremely low selectivity of a UV detector operated at 210 nm. Compounds with conjugated double bonds, for instance phenolics and nucleotide phosphates, because of their strong UV absorption, show pronounced signals even at low concentrations. Such compounds may cause severe problems for quantification of some organic acids, particularly those with a low capacity factor like tartaric and malic acid. Efforts have been made to remove interfering compounds using custom-made anion exchange columns [12,27] but that requires handling of huge volumes and has thus not found broad application although promising results were obtained.

Here we use commercial solid NH2 solid phase extraction (SPE) columns for sample preparation. Under the conditions applied, sugars appear in the flow through while organic acids are well retained. Thus, the flow through is essentially free of organic acids and other compounds binding strongly to NH2 phases and can be used for quantification of sugars with amino columns and RI detection. The organic acids bound to the SPE columns are eluted with phosphoric acid and analysed by HPLC using a C18 column and detection by UV absorption at 210 nm. Including SPE enhances selectivity considerably since only acidic compounds are retained by the SPE column, while many UV absorbing compounds like phenolics are efficiently removed. It is also possible to elute the organic acids with trifluoroacetic acid, derivatise them by methylation and analyse the formed volatile methyl esters by GC–MS (see Supplementary Methods).

Lactose is added as internal standard for sugars and tricarballylic acid for organic acid to the samples. Both compounds are usually absent from tomato and other fruits. The use of internal standards compensates for losses during sample preparation and detector drift and renders precise volume control unnecessary except for pipetting of the sample, making the methods simple and highly reproducible.

## Solutions

Tricarballylic acid 10 g/l (dissolve 500.0 mg tricarballylic acid in water to a total volume of 50.0 ml; keep at −20 °C)

Lactose 100 g/l (dissolve 5263 mg lactose monohydrate in water to a total volume of 50.0 ml; keep at −20 °C)

Sugars standard mix: glucose 40 g/l, fructose 40 g/l, sucrose 40 g/l (dissolve 2000 mg fructose, 2000 mg glucose water free (or 2200 mg glucose monohydrate) and 2000 mg sucrose in water to a total volume of 50.0 ml; keep at −20 °C) *Note: if only cultivated tomatoes shall be analysed sucrose can be omitted.*

Organic acids standard mix: citric acid 5 g/l, malic acid 1 g/l (dissolve 500.0 mg citric acid water free and 100.0 mg malic acid in water to a total volume of 100.0 ml; keep at −20 °C) *Note: citric and malic acid are the main organic acids in tomato. Other organic acids may be added as well. Commercial malic acid usually contains small amounts of fumaric acid. Due to the high UV absorption coefficient of fumaric acid an additional peak is usually visible in standards containing malic acid.*

Acetonitrile (ACN) 100%, HPLC grade

ACN 90% (mix 225 ml ACN 100% with 25 ml water)

ACN 40% (mix 100 ml ACN 100% with 150 ml water)

Phosphoric acid 4 mol/l (add 27.4 ml phosphoric acid 85% to approximately 60 ml water, mix well, let cool to room temperature and add water to a total volume of 100 ml)

Phosphoric acid 400 mmol/l (mix 10 ml phosphoric acid 4 mol/l with 90 ml water)

Ammonia solution 1 mol/l (add 7.5 ml ammonia solution 25% to approximately 80 ml water, mix well and add water to a total volume of 100 ml)

Eluent A for organic acids: 20 mM ammonium phosphate pH 2.6 (mix 25 ml phosphoric acid 4 mol/l with approximately 4700 ml water and set the pH to 2.6 with ammonia solution 1 mol/l. Add water to a total volume of 5000 ml and filter using a 0.22 μm nylon or PTFE membrane filter. The solution can be kept at room temperature for up to 2 months)

Eluent B for organic acids: 20 mM ammonium phosphate pH 2.6 in 10% ACN (mix 25 ml phosphoric acid 4 mol/l with approximately 4600 ml water and 100 ml ACN 100% and set the pH to 2.6 with ammonia solution 1 mol/l. Add water to a total volume of 5000 ml and filter using a 0.22 μm nylon or PTFE membrane filter)

Eluent for sugars: ACN 80% (mix 1553.5 g ACN 100% with 498.5 g water)

## Materials

Miracloth

Chromabond NH_2_ 100 mg columns (Macherey-Nagel, Düren, Germany; cat. no. 730,031)

Solid phase extraction apparatus

Ultra-Turrax or Warring Blender homogeniser

Nucleodur 100-5 NH2 125 × 4 mm column (Macherey-Nagel, Düren, Germany)

Nucleodur 100-5 NH2 4 × 3 mm precolumn (Macherey Nagel)

Nucleodur 100-5 C18ec 250 × 4 mm column (Macherey Nagel)

Nucleodur 100-5 C18ec 4 × 3 mm precolumn (Macherey Nagel)

## Analytical instrumentation

An isocratic HPLC system equipped with a RI detector can be used for analysis of sugars. A binary high pressure gradient HPLC system equipped with a UV detector should be used for quantification of organic acids. It is also possible to use a low pressure gradient system but that requires adaptation of the elution gradient and increases the analysis time. In this study, the HPLC system used for analysis of both sugars and organic acids consisted of a SCL-10 A system controller, two Shimadzu LC-10ADvp pumps each equipped with a degasser and a FCV-10AL valve for eluent selection, a SIL-10 A autosampler, a CTO-10ASvp column oven, a SPD-10 A UV detector and an Agilent 1047 A RI detector. Chromatograms were evaluated with the Clarity software package (DataApex, Prague, Czech Republic).

## Protocol

### Sample preparation

1 Homogenise approximately 100 g tomato fruits with an Ultra-Turrax or a Warring Blender homogeniser.
2 Transfer the homogenate into 50 ml tubes and centrifuge at 4000*g* for 5 min.
3 Filter through Miracloth. *Note: paper filters may be used as well but filtration is more rapid with Miracloth.*
4 Filter approximately 2 ml of the filtrate through a 0.22 μm membrane syringe filter (PP, nylon or hydrophilic PTFE membranes are suitable) and use the filtrate for SPE. *Note: the filtrate can be stored at −20C° until analysis.*
5 Using a volumetric pipet transfer exactly 25 ml of the filtrate into a beaker placed on an analytical balance and weigh the filtrate. Calculate the density of the supernatant by dividing the weight by the volume. *Note: the density is required for conversion of the concentration of the sugars and organic acids from g/l to the content in g/kg. However, if only the concentration (g/l) shall be calculated this step can be omitted.*

### Solid phase extraction (SPE)

1 Transfer exactly 400 μl extract into a 1.5 ml reaction tube and add 100 μl tricarballylic acid 10 g/l (internal standard for organic acids), 100 μl lactose 100 g/l (internal standard IS for sugars) and 400 μl ACN 100%.
2 Centrifuge the sample at >10,000 rpm for 5 min.
3 Equilibrate a Chromabond 100 mg NH2 column with 1 ml ACN 100% and subsequently with 1 ml ACN 40%.
4 Remove the collection tube containing the effluent and replace by a new tube.
5 Load the diluted sample (1000 μl) onto the equilibrated SPE column and let flow through by gravity.
6 Wash with 1 ml ACN 40%.
7 Apply vacuum (400 mbar) for 30 s.
8 Remove the collection tube containing the combined flow through and wash. This solution is directly used for quantification of sugars and can be stored for up to one month in the dark at room temperature.
9 Place a new collection tube in the SPE apparatus.
10 Load 1000 μl 400 mM phosphoric acid (for analysis of organic acids by HPLC) or 1000 μl 400 mM trifluoroacetic acid (for analysis of organic acids by GC–MS; see Supplementary Methods) onto the column and apply a gentle vacuum (approx. 700 mbar) to allow entering the solution into the column.
11 Let the eluent run through the column by gravity.
12 Finally, apply vacuum (400 mbar) to collect the whole eluate. The eluate can be used directly for quantification of organic acids by HPLC. It is possible to store the eluate at −20 °C for up to 1 month prior to analysis by HPLC.

### Analysis of sugars by HPLC

1 Transfer 200 μl flow-through into an autosampler vial and add 800 μl ACN 90%. Close the lid and mix well. Note: at a temperature of less than 25 °C phase separation may occur and microdroplets containing high sugar concentrations may separate, which would introduce errors into the quantification of sugars. Thus, it is important to work at a sufficient room temperature or to prewarm the solutions in an incubator.
2 Prepare standards by mixing the compounds as indicated in Table 1.

**Table 1:** Standards for quantification of sugars.

No.	Sugar standard mix μl	Lactose μl	H_2_O μl	ACN 100% μl
St 1	0	100	1400	1000
St 2	40	100	1360	1000
St 3	80	100	1320	1000
St 4	120	100	1280	1000
St 5	160	100	1240	1000
St 6	200	100	1200	1000
St 7	300	100	1100	1000
St 8	400	100	1000	1000
3 Transfer 200 μl of the standards into autosampler vials and add 800 μl ACN 90%. Close the lid and mix well. Note: the temperature must be kept above 25 °C; see comment to step 1 for details.
4 Analyse the standards and samples by HPLC using a Nucleodur 100-5 NH2 125 × 4 mm column preceded by a Nucleodur 100-5 NH2 4 × 3 mm precolumn. Eluent for sugars (80% ACN) is used at a flow rate of 1.0 ml/min. The column oven temperature is set to 30 °C. Full loop injection is applied using a 100 μl sample loop. For detection a RI detector is used. The analysis time is typically 18 min. Typical chromatograms are shown in Fig. 1. For evaluation the ratio of the areas of the analyte and the internal standard are plotted against the ratios of the masses (Fig. 1D).

**Fig. 1:**
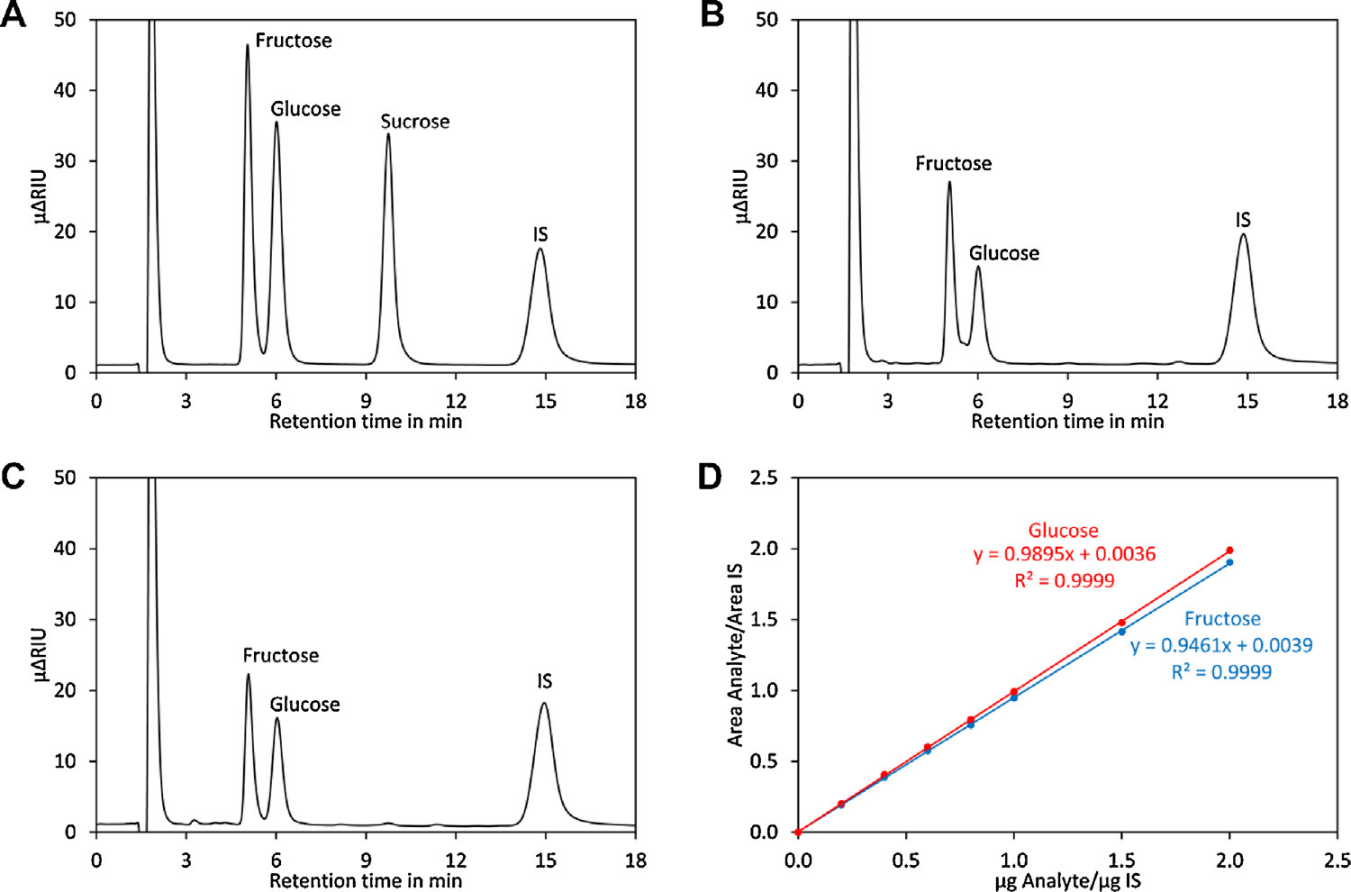
Analysis of sugars by HPLC. (A) Chromatogram of a standard containing the indicated sugars. (B) Chromatogram for ripe Heinz 1706 tomatoes. (C) Chromatogram for green, unripe Heinz 1706 tomatoes. (D) Calibration curves for glucose (red) and fructose (blue).

### Analysis of organic acids by RP-HPLC

1 Transfer the eluate into an autosampler vial and close the lid. Note: only eluates obtained with 400 mM phosphoric acid can be used for RP-HPLC.
2 Prepare standards in autosampler vials according to Table 2.

**Table 2:** Standards for quantification of organic acids.

No.	Organic acid standard mix in μl	Tricarballylic acid in μl	Water in μl	Phosphoric acid^a^ 4 mol/l in μl
St 1	0	100	800	100
St 2	20	100	780	100
St 3	50	100	750	100
St 4	100	100	700	100
St 5	200	100	600	100
St 6	400	100	400	100
St 7	600	100	200	100
St 8	800	100	0	100

a For quantification of organic acids by GC–MS phosphoric acid must be replaced by TFA.
3 Analyse the standards and samples by HPLC using a Nucleodur 100-5 C18ec 250 × 4 mm column preceded by a Nucleodur 100-5 C18ec 4 × 3 mm precolumn. The injection volume is set to 25 μl and the column oven temperature to 25 °C. Gradient elution is performed according to Table 3 at a flow rate of 0.6 ml/min. The acids are separated within the first 16 min of the gradient while the steps afterwards are required for flushing and re-equilibration of the column. For detection a UV detector operated at 210 nm is used. Typical RP-HPLC chromatograms are shown in Fig. 2. The data are evaluated as described for analysis of sugars.

**Table 3:** Gradient used for elution of organic acids.

Time in min	Eluent A in %	Eluent B in %
0	100	0
16	62.5	32.5
17	0	100
18	100	0
25	100	0

**Fig. 2:**
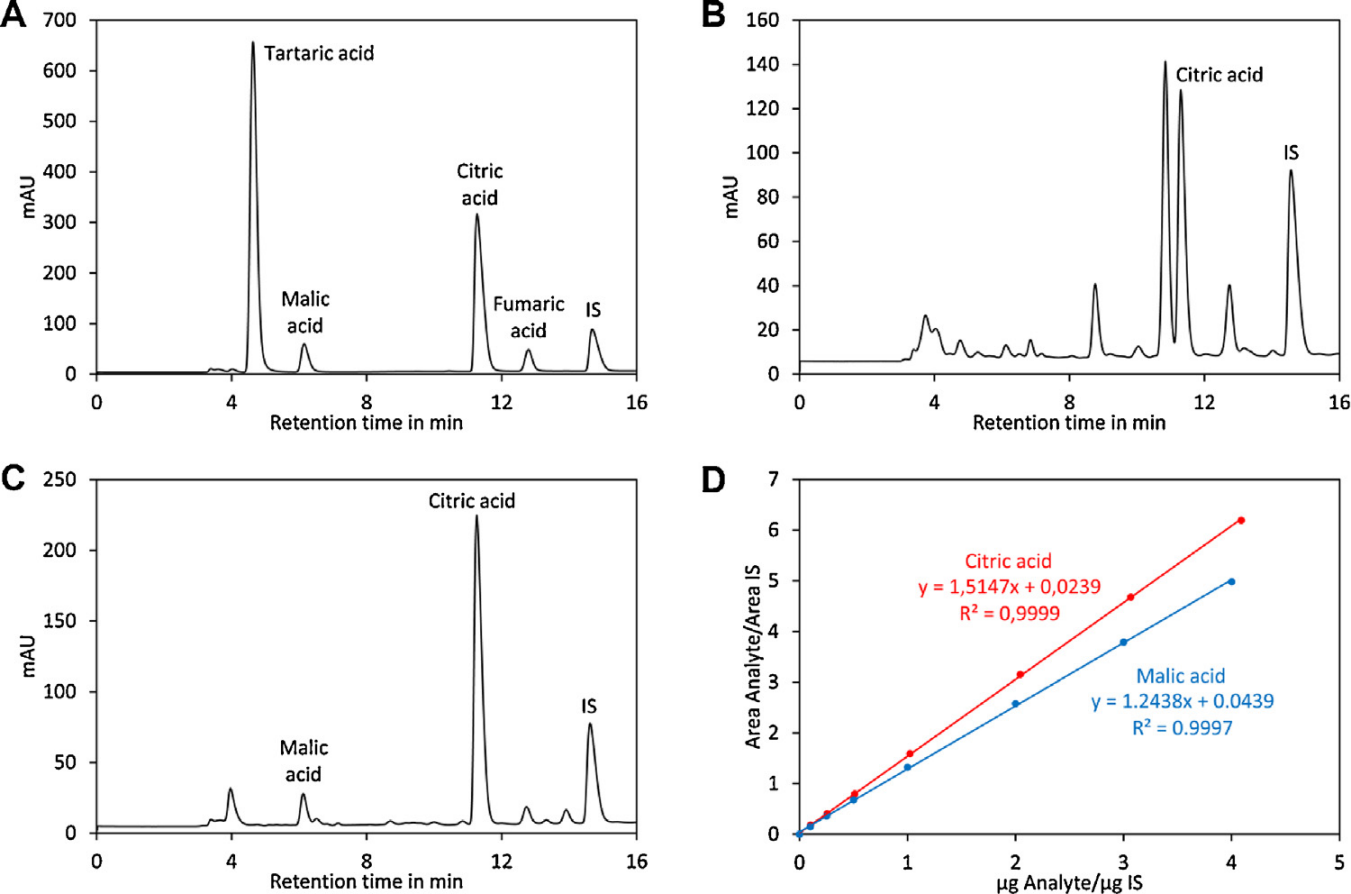
Analysis of organic acids by HPLC. (A) Chromatogram of a standard containing citric acid, malic acid and tartaric acid. The fumaric acid peak visible at 12.7 min originates from an impurity in malic acid. (**B**) Chromatogram for ripe Heinz 1706 tomatoes. (C) Chromatogram for green, unripe Heinz 1706 tomatoes. (D) Calibration curves for citric acid (red) and malic acid (blue).

## Additional information

### Validation of the method

**Fig. 3:**
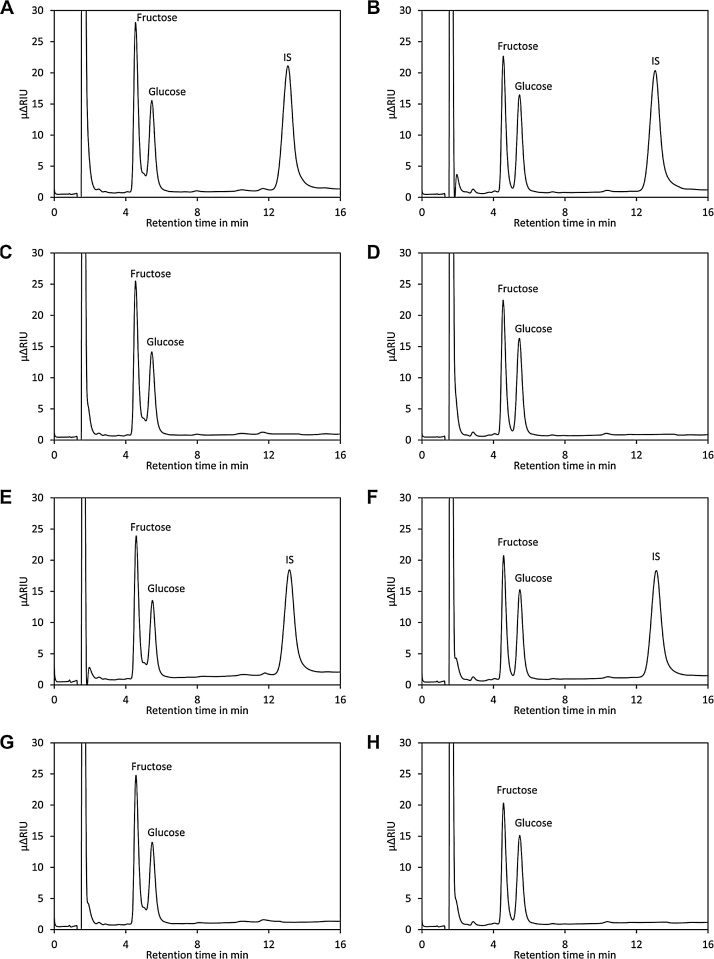
Effect of SPE on analysis of sugars by HPLC. (A) Chromatogram of a sample of ripe Heinz 1706 tomato spiked with lactose as internal standard (IS). (B) Same as (A) but green, unripe Heinz 1706 tomato was used. (C) Same as (A) but no internal standard was added. (D) Same as (B) but addition of the internal standard was omitted. (E) The same sample as in (A) was injected without prior purification by SPE. (F) The same sample as in (B) was injected without prior purification by SPE. (G) The same sample as in (C) was injected without prior purification by SPE. (H) The same sample as in (D) was injected without prior purification by SPE.

Sugars and organic acids are major factors for the flavour of tomatoes. Sugars are frequently quantified by HPLC using amino-columns and RI detection. RI detection is relatively insensitive, but this is not a critical issue for analysis of tomatoes and many other fruits. Importantly, interference from other constituents is unlikely since the RI detector shows a similar response for most compounds. Using an appropriate dilution ensures that only major constituents are detected. Accordingly, application of SPE has little impact on the appearance of the chromatogram as indicated in Fig. 3. However, SPE with an NH2 column has the advantage that compounds binding irreversibly to the NH2 stationary phase are efficiently removed and thus contamination of the analytical column is minimised.

Aliphatic carboxylic acids lack strong chromophores and a wavelength of 210 nm must be used for their detection. At such a low wave length many compounds absorb strongly and thus interference with quantification of organic acids is frequently observed. Most of these interfering compounds are neutral molecules like phenolic compounds.

**Fig. 4:**
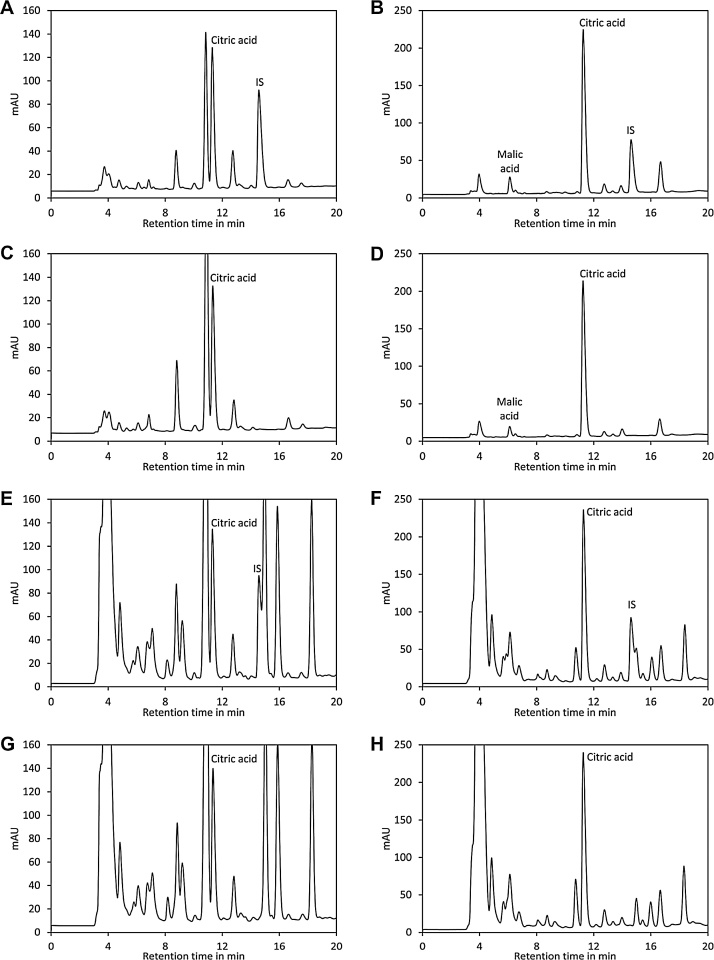
Effect of SPE on analysis of organic acids by HPLC. (A) Chromatogram of a sample of ripe Heinz 1706 tomato spiked with tricarballylic acid as internal standard (IS). (B) Same as (A) but green, unripe Heinz 1706 tomato was used. (C) Same as (A) but no internal standard was added. (D) Same as (B) but addition of the internal standard was omitted. (E) The same sample as in (A) was injected without prior purification by SPE. (F) The same sample as in (B) was injected without prior purification by SPE. (G) The same sample as in (C) was injected without prior purification by SPE. (H) The same sample as in (D) was injected without prior purification by SPE.

Here we applied solid phase extraction with NH2 columns for removal of non-acidic compounds. To investigate the efficiency of SPE we analysed samples of ripe and unripe Heinz 1706 tomatoes with and without purification by SPE. Analysis by HPLC revealed a quite clean chromatogram for samples after SPE (Fig. 4A–D) while many peaks were visible in raw samples (Fig. 4E–H). The citric acid peak was visible in samples with and without SPE while the malic acid peak could only be reliably identified in samples after SPE. At the retention time of the internal standard (tricarballylic acid) no peak was visible in samples of ripe and unripe tomato (Fig. 4C and D) after SPE. In contrast, there were interfering peaks at that retention time in samples without SPE. Thus, SPE with NH2 columns is a simple and efficient way for removal of interfering compounds for quantification of organic acids in tomato. In addition, the flow through can be used for quantification of sugars. In this case, SPE has the advantage that compounds binding strongly to NH2 columns are removed, thereby preventing contamination of the analytical column.

To investigate the recovery rate of SPE we prepared a test mixture containing 5000 mg/l of each malic, citric, tartaric and tricarballylic acid and 25 g/l each of glucose, fructose, sucrose and lactose. Analysis of the sugars in the flow-through showed excellent recovery rates for the four tested sugars of more than 98% (Table 4).

**Table 4:** Recovery rate for sugars (flow through).

Compound	Repeats	Recovery rate in %	SD
Fructose	4	99.3	0.5
Glucose	4	99.2	0.3
Sucrose	4	99.4	0.4
Lactose (IS)	4	98.8	0.9

To assess the recovery rates of organic acids they were eluted with either 400 mM phosphoric acid for analysis by HPLC or with 400 mM TFA for quantification by GC–MS. With both methods very high recovery rates in the range of 95% were obtained (Table 5). Importantly, the recovery rates for all tested organics acids were equal, indicating that tricarballylic acid is a suitable internal standard.

**Table 5:** Recovery rate for organic acids (eluate).

Compound	Repeats	Elution with 400 mM H_3_PO_4_		Elution with 400 mM TFA	
		Recovery rate in %	SD	Recovery rate in %	SD
Citric acid	4	95.9	1.8	97.1	2.2
Malic acid	4	95.1	1.8	96.4	6.4
Tartaric acid	4	95.3	1.7	93.4	6.0
Tricarballylic acid (IS)	4	95.6	2.0	94.6	5.2

To assess reproducibility of the method an extract obtained from ripe Heinz 1706 tomatoes was prepared, aliquoted and analysed in quadruplicate on four consecutive days (Table 6). The results for the individual days (intra-day) ranged from 12.81 g/l to 13.33 g/l for glucose and 8.50 g/l to 9.36 g/l for fructose with standard deviations (SD) ranging from 0.9% to 5.7%. The overall (inter-day) result for glucose and fructose were 13.02 g/l (SD: 3.5%) and 8.95 g/l (SD: 4.2%), respectively.

**Table 6:** Intra- and inter-day repeatability for quantification of sugars in tomato.

Experiment	Repeats	Glucose			Fructose		
		Average in g/l	SD in g/l	SD in %	Average in g/l	SD in g/l	SD in %
Day 1	4	12.81	0.35	2.7	9.00	0.20	2.2
Day 2	4	13.33	0.14	1.0	9.36	0.09	0.9
Day 3	4	13.03	0.38	2.9	8.96	0.13	1.4
Day 4	4	12.89	0.74	5.7	8.50	0.39	4.6
Inter-day	16	13.02	0.46	3.5	8.95	0.38	4.2

Also analysis of citric acid in ripe tomato by HPLC yielded highly reproducible values (Table 7). The intra-day results ranged for HPLC from 1901 mg/l to 1974 mg/l with SDs from 1.1% to 2.1% and an overall result of 1940 mg/l with an SD of 2.2%.

**Table 7:** Intra- and inter-day repeatability for quantification of citric acid in tomato.

Experiment	HPLC				GC-MS			
	Repeats	Average in mg/l	SD in mg/l	SD in %	Repeats	Average in mg/l	SD in mg/l	SD in %
Day 1	4	1924	40	2.1	4	1978	20	1.0
Day 2	4	1974	21	1.1	4	2097	71	3.4
Day 3	4	1901	21	1.1	4	1911	59	3.1
Day 4	4	1961	42	2.1	4	1998	59	2.9
Inter-day	16	1940	42	2.2	16	1996	85	4.3

To confirm these results by an independent method the citric acid content of the same sample was also quantified by GC–MS as described in the supplementary data section. Importantly, highly similar vales were obtained by HPLC (1940 mg/l) and GC–MS (1996 mg/l; Table 7) with a difference of less than 3%. In addition, citric and malic acid were also quantified by HPLC and GC–MS in green tomato (Table 8). For malic acid, slightly lower values were obtained by HPLC than by GC–MS. However, it must be mentioned that GC–MS is very sensitive for citric and tricarballylic acid while it is less suitable for quantification of malic acid as indicated by the low detector response (Supplementary Fig. 1), which may explain the deviation for malic acid. For citric acid, almost identical values (less than 1% difference) were obtained by HPLC and GC–MS.

**Table 8:** Intra-day repeatability for quantification of citric acid in tomato.

Compound	HPLC				GC-MS			
	Repeats	Average in mg/l	SD in mg/l	SD in %	Repeats	Average in mg/l	SD in mg/l	SD in %
Citric acid	4	4553	145	3.2	4	4526	145	3.2
Malic acid	4	511	15	2.9	4	607	15	2.4

## Comparison with other HPLC-based methods

Quantification of sugars in tomato by HPLC in a simple and reliable technique that is widely applied (Table 9). In several publications sample preparation is limited to filtration [28,17,23]. To minimise contamination of the column solid phase extraction with C18 columns has been used [12,13]. This is an excellent method for removal of lipophilic compounds like pigments, which bind strongly to the SPE column, while the hydrophilic sugars pass the SPE column and appear in the flow through and washing solution. However, a disadvantage of this method is that removal of organic acids may be incomplete and that another method for sample preparation must be used for organic acids. Here we used NH2 solid phase extraction columns. Similar to C18 columns the sugars appear in the flow through and wash. However, in contrast to C18 columns, the organic acids are quantitatively retained on the NH2 columns. The organic acids can subsequently be eluted and analysed by HPLC. Thus, the method proposed here requires only one SPE extraction for analysis of both sugars and organic acids. For separation of sugars polar stationary phases are frequently used in combination with eluents containing high levels or organic solvents, mainly ACN [12]. Alternatively, special cation exchange columns in the Ca form can be used [23,28]. The latter has the advantage that pure water can be used as solvent but the disadvantages that the sample should be free from calcium and that the required columns are extremely expensive.

**Table 9:** Methods for quantification of sugars in tomato by HPLC.

Reference	Sample	Column;	Eluent	Detection	Repeatability^b^	
	preparation	column oven temperature^a^			intra-day	inter-day
This study	NH2 SPE column	Nucleodur NH2 5 μm 250 × 4.6 mm; 30 °C	ACN/H_2_O = 80/20	RI	F: 2 G: 3	F: 4 G: 4
Gancedo and Luh [12]	SEP PAC C18 SPE column	Micro-Bondapak carbohydrate 4 300 × 4 mm; RT	ACN/H_2_O = 80/20	RI	nr	nr
Yelle et al. [13]	C18 SPE column	P/10 carbohydrate 250 × 4.6 mm; nr	MeOH/H_2_O/ NH_4_OH = 79/20/1	RI	nr	nr
Vermeir et al. [17]	–	Aminex HPX-87C; 80 °C	H_2_O	RI	F: 9^c^ G: 11^c^	nr
Saito et al. Saito et al. [28]	–	TSK-GEL Amide-80 250 × 4.6 mm; 80 °C	ACN/H2O = 75/25	RI	nr	nr
Zushi and Matsuzone [23]	–	Shim-pack SCR-101C 300 × 7.9 mm; 80 °C	H_2_O	RI	nr	nr

a RT, room temperature; nr: not reported.

b The repeatability is reported as relative SD in %. F, repeatability for fructose; G: repeatability for glucose; nr, not reported.

c The reported levels are unusually small: 2.00–2.43 g/l for glucose and 1.81–2.46 g/l for glucose.

For detection all studies listed in Table 9 used a refractive index (RI) detector. This type of detector is ideal for detection sugars since they are present in tomatoes in high concentrations and thus the low sensitivity of the RI detector is not a limiting factor.

Sample preparation for analysis of organic acids (Table 10) includes often SPE with an anion exchange resin [12,27]. Also the NH2 columns used in this study act by the same mechanism: in aqueous systems stationary NH2 phases are protonated and have considerable anion exchange capacity allowing their use for retaining or separation anions like organic acids [29]. Two other studies ([17,23]) did not use any sample preparation apart from filtration. However, no chromatograms were shown in these studies and thus the quality of separation is difficult to evaluate. In the study of [17] the same samples were also measured by enzymatic assays, which might be seen as a confirmation. However, the reported values for citric acid are very small, 0.36-0.55 g/l, which is approximately 10 times lower than in other publications, making assessment of the results difficult. Typical results for citric acid in tomato fruits are 5.41–8.06 g/kg [11], 5.00–10.00 g/l [16], 3.94–7.11 g/l [15] and 1.94–4.55 g/l (this study).

**Table 10:** Methods for quantification of organic acids in tomato by HPLC.

Reference	Sample	Column;	Eluent^b^	Detection^c^	Repeatability^d^	Results	
	preparation	column oven temperature^a^			intra-day	inter-day	confirmed by another method
This study	SPE with NH2 columns	Nucleodur C18ec, 5 μm, 250× 4.6 mm; 25 °C	Gradient, 10 mM AP pH 2.6, ACN	UV, 210 nm	C: 2	C: 2	Yes^e^
Marconi et al. [27]	Amberlite IRA-400	Alltima C18, 5 μm, 250 × 4.6 mm; 50 °C	H_2_O/MeOH/TFA = 97.7/2.2/0.1	UV, 210 nm	C: 2	nr	No
Gancedo and Luh [12]	Dowex 1-X8 and SEP PAC C18	Aminex HPX-87 ion exclusion; 60 °C	10 mM H_2_SO_4_	RI	nr	nr	No
Vermeir et al. [17]	–	Prevail org. acids column 250 × 4.6 mm; nr	H_2_O adjusted to pH 2.5 with formic acid	UV, 200 nm	C: 11	nr	Yes^f^
Zushi and Matsuzone [23]	–	Shim-pack SCR-102H 300 × 7.9 mm; 40 °C	5 mM p-toluene-sulfonic acid	CD^g^	nr	nr	No

a nr: not reported.

b AP, ammonium phosphate buffer.

c RI, refractive index detector; CD, conductivity detector.

d The repeatability is reported as relative SD in %. C, repeatability for citric acid; nr, not reported.

e GC–MS.

f Enzymatic assay. The reported values for citric acid are with 0.36-0.55 g/l unusually small.

g Conductivity detection after post-column mixing with 20 mM bis-TRIS and 100 μM EDTA in 5 mM p-toluenesulfonic acid.

Organic acids are often separated by reversed phase chromatography ([27,17]) or by ion exclusion chromatography ([12,23]). For detection short wave UV in the range of 200 nm–210 nm, RI or conductivity is employed (Table 10). Conductivity detection has a higher selectivity than UV and RI detection, since only ionic compounds are detected. However, prior detection the eluent stream must be post-column mixed with a buffer to increase the pH to convert the organic acids to their ionised form [23], which makes the HPLC system more complicated. The disadvantage of low selectivity by UV detection can be compensated by application of SPE, as indicated by Fig. 4 and the excellent repeatability of such methods (Table 10).

## Conclusion

The methods described here allow reliable quantification of glucose, fructose and non-volatile organic acids in tomato and presumably in other fruits. The main advantage compared to previous studies using similar methods is the application of commercially available NH2 SPE columns that allow efficient purification of the organic acids. This enhances selectivity for organic acid analysis, an important advantage for RP-HPLC. For GC–MS sample preparation by NH2 SPE columns has the advantage that sugars, which would otherwise contaminate the injector, are efficiently removed. For HPLC of sugars the described SPE method minimises contamination of the column that may otherwise lower the lifetime of the analytical HPLC column.

## Conflict of interests

The authors declare to have no conflict of interests.
