# Supplementary material for: Quantification of sugars and organic acids in tomato fruits
Source: MethodsX. 2018 May 25;5:537–50. doi: 10.1016/j.mex.2018.05.014 (PMC6046607; doi:10.1016/j.mex.2018.05.014)
Supplement: Supplementary file 1 [file mmc1.pdf]

Supplementary methods: analysis of organic acids by GC-MS

Solutions

Trifluoroacetic acid, 400 mM (mix 3.1 ml trifluoroacetic acid with water to a final volume of 100 ml)
Trifluoroacetic acid, 4 M (mix 3.1 ml trifluoroacetic acid with water to a final volume of 10 ml)
Methanol, HPLC grade
Acetic acid, 98-100%
(Trimethylsilyl)diazomethane solution 2 M in diethylether (commercially available for instance from Sigma Aldrich, Fisher Scientific or Acros Organics)
Derivatisation reagent (mix 900 µl methanol with 300 µl (trimethylsilyl)diazomethane solution. The reagent must be prepared immediately before use and handled in an efficient fume hood)
Acetic acid, 1 mol/l in methanol (mix 945 µl methanol with 55 µl acetic acid. The reagent must be prepared immediately before use)

Materials

Vacuum concentrator
VF-5ms 30 m x 0.25 mm x 0.25 µm (Agilent Technologies, Santa Clara, CA, USA)

Analytical instrumentation

A Varian 431-GC gas chromatograph equipped with a CP-8400 autosampler and connected to a 210-MS mass spectrometer was used.

Protocol

1. Prepare standards according to Table 2 in 1.5 ml reaction tubes but replace phosphoric acid 4 mol/l by trifluoroacetic acid (TFA) 4 M. *Note: only eluates obtained with 400 mM trifluoroactic acid can be used for GC-MS.*
2. Transfer 10 µl eluate or standard into a 1.5 ml reaction tube and evaporate in a vacuum concentrator to complete dryness.
3. Add 60 µl derivatisation reagent and mix well. The yellow colour of the reagent must remain. If not add more reagent.
4. Incubate in a thermomixer set to 25°C and 800 rpm for 5 min prior addition of 5 µl 1 M acetic acid in methanol. The yellow colour must disappear. If not add more methanolic acetic acid.
5. Centrifuge the tubes at >10000 g for 5 min.
6. Transfer 50 µl into autosampler vials.
7. Analyse the samples by GC-MS using a VF-5ms 30 m x 0.25 mm x 0.25 µm capillary column and helium as carrier gas at a flow rate of 1 ml/min and a split ratio of 1:10. The injector is set to 220°C. Injection is performed in the splitless mode. The temperature program is set according to Supplementary Table 1. The transfer line is operated at 190°C, the ion trap at 160°C and the manifold at 40°C. MS spectra are recorded from 5 to 12 min and from m/z 50 to 250. Typical chromatograms and MS spectra are shown in Supplementary Figure S1 and Supplementary Figure S2, respectively. For evaluation the sum of the intensities of the ion listed in Supplementary Table 2 should be used because the intensities of the ions are somehow dependent on the on-column amount of the compound with higher m/z being more pronounced with high amounts and lower m/z being more intense at low amounts.

Supplementary Table 1: Column oven program for GC-MS.

Time in min	Temperature in °C	Heating rate in °C/min
0	45	0
1	45	15
11	210	30
15	300	-

Supplementary Table 2: Ion m/z for quantification of organic acids as their methyl esters.

Compound	Retention time in min	m/z[a]
Citric acid	10.4	235 ($[M+H]^+$), 143, 101
Malic acid	7.2	163 ($[M+H]^+$), 103, 43
Tartaric acid	8.2	179 ($[M+H]^+$), 119
Tricarballylic acid (IS)	9.8	219 ($[M+H]^+$), 187, 127, 126, 99

[a] The protonated, positively charged molecule ion is indicated by $[M+H]^+$ in brackets. Typical MS spectra are shown in Supplementary Figure 2.

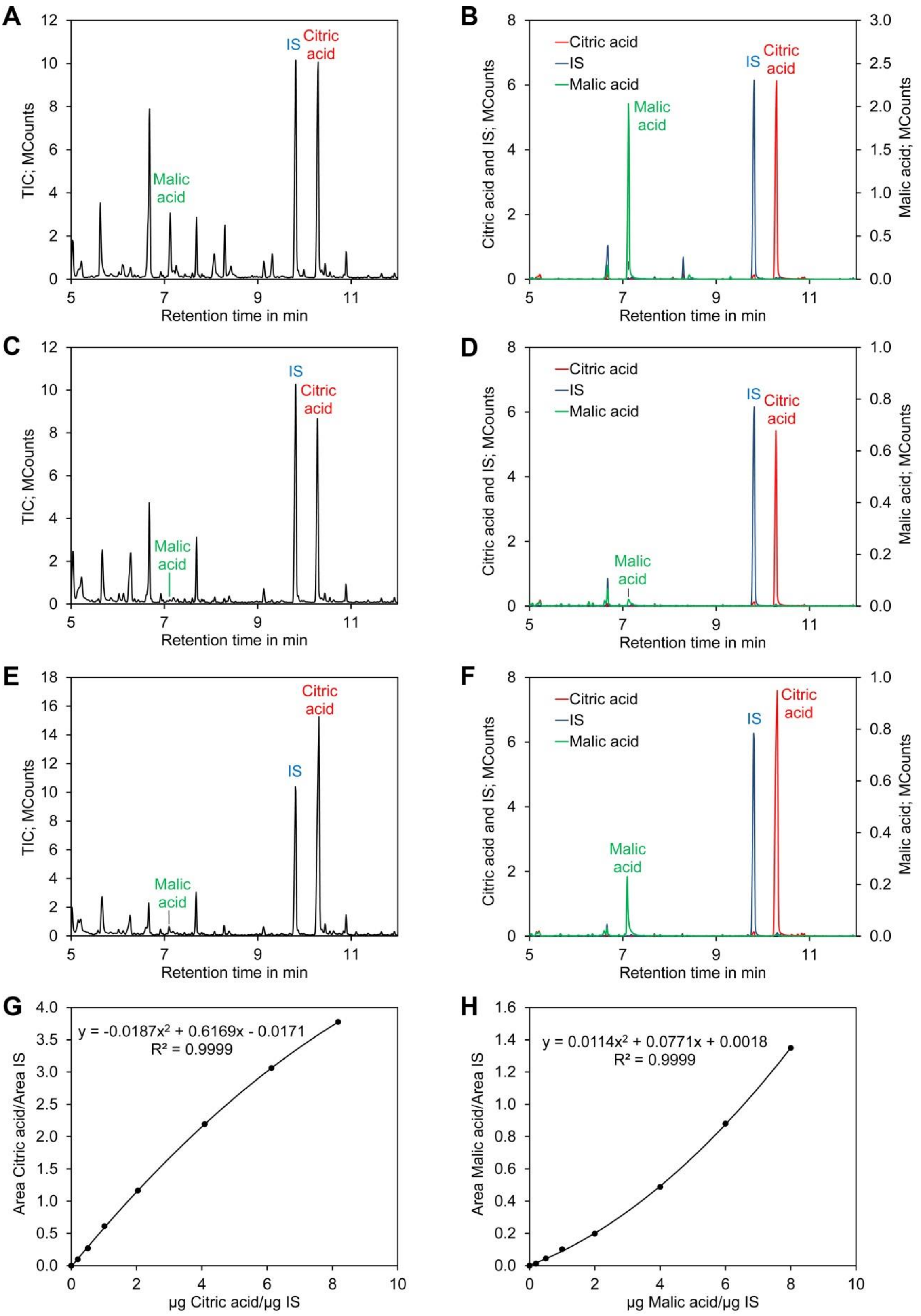

Supplementary Figure 1: Analysis of organic acids by GC-MS. (**A**) Total ion chromatogram (TIC) of a standard containing 2500 mg/l of each citric acid and malic and 1250 mg/l tricarballylic acid. (**B**) Same sample as in (A) but selected ion chromatograms for citric acid (red; m/z 235, 143 and 101), malic acid (green; m/z 163 and 103) and the internal standard (tricarballylic acid, blue; m/z 219, 187, 127, 126 and 99). (**C**) TIC obtained with a sample of ripe Heinz 1706 tomatoes and (**D**) the corresponding selected ion chromatograms. (**E**) TIC for green, unripe Heinz 1706 tomatoes and (**F**) the corresponding selected ion chromatograms. (**G**) Calibration curve for citric acid. (**H**) Calibration curve for malic acid.

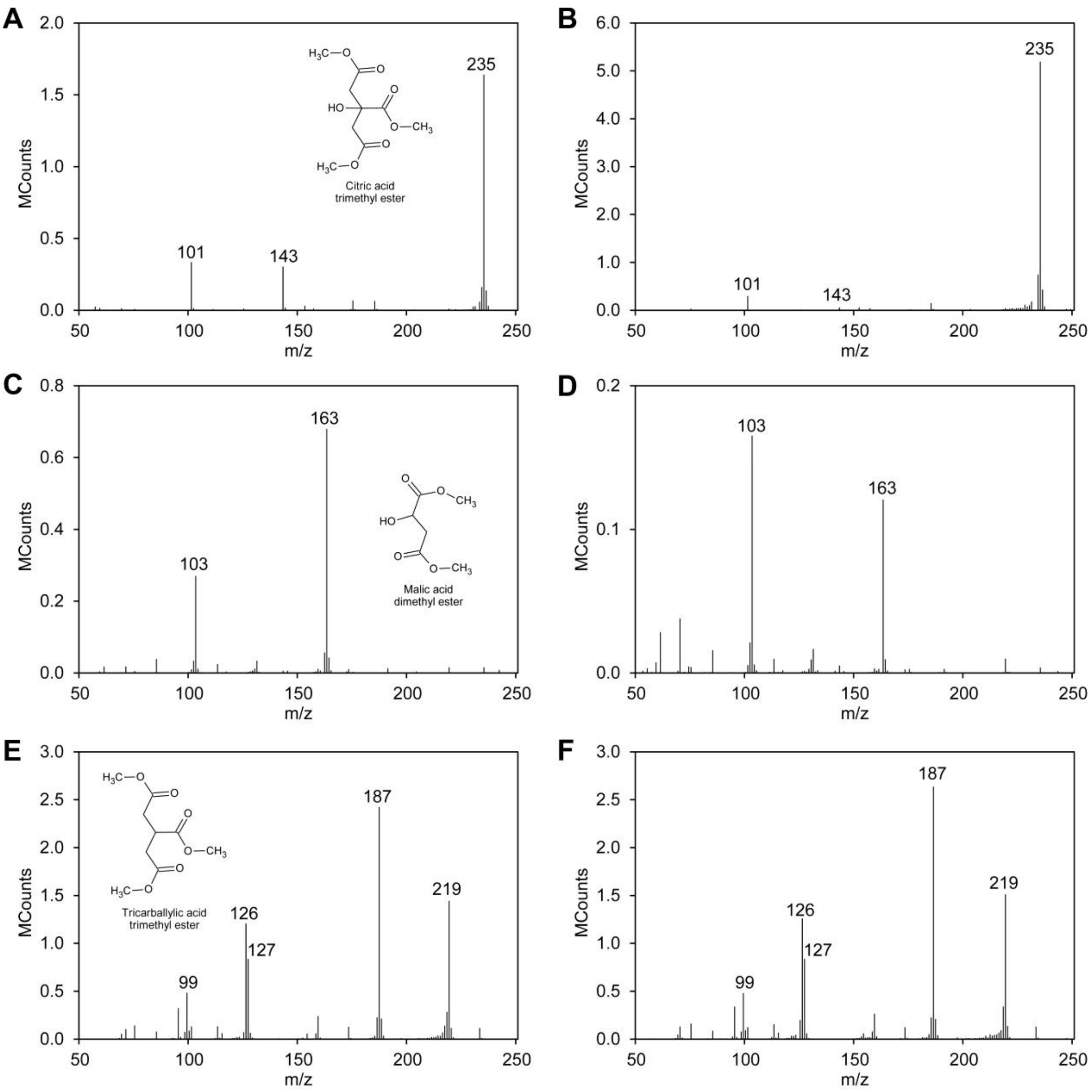

Supplementary Figure S2: Mass spectra of organic acids as methyl esters. (**A**) MS of the citric acid peak of a standard containing 639 mg/l citric acid. (**B**) MS of the citric acid peak observed in red Heinz 1706 tomato containing approximately 2000 mg/l citric acid. (**C**) MS of the malic acid peak of a standard containing 625 mg/l malic acid. (**D**) MS of the malic acid peak of green Heinz 1706 tomato containing approximately 500 mg/l malic acid. (**E**) MS of the internal standard (tricarballylic acid) peak of a standard containing 1250 mg/l tricarballylic acid. (**F**) MS of the internal standard peak of a sample spiked with tricarballylic acid to a level of 1250 mg/l.
